# Parental Influence on Young Children's Physical Activity

**DOI:** 10.1155/2010/468526

**Published:** 2010-07-07

**Authors:** Cheryl A. Zecevic, Line Tremblay, Tanya Lovsin, Lariviere Michel

**Affiliations:** ^1^Department of Human Kinetics, Laurentian University, Sundbury, ON, Canada P3E 2C6; ^2^Department of Psychology/School of Medicine, Laurentian University, Ramsey Lake Road, Sudbury, ON, Canada P3E 2C6; ^3^Department of Psychology, Laurentian University, Sundbury, ON, Canada P3E 2C6; ^4^Department of Human Kinetics/School of Medicine, Laurentian University, Sundbury, ON, Canada P3E 2C6

## Abstract

Parents influence on their young children's physical activity (PA) behaviours was examined in a sample of 102 preschool-aged children (54 boys). Questionnaires regarding family sociodemographics and physical activity habits were completed. Results showed that children who received greater parental support for activity (*B* = .78, *P* < .10) and had parents who rated PA as highly enjoyable (*B* = .69, *P* < .05) were significantly more likely to engage in one hour or more of daily PA. Being an older child (*B* = −.08, *P* < .01), having older parents (*B* = −.26, *P* < .01), and watching more than one hour of television/videos per day (*B* = 1.55, *P* < .01) reduced the likelihood that a child would be rated as highly active. Children who received greater parental support for PA were 6.3 times more likely to be highly active than inactive (*B* = 1.44, *P* < .05). Thus, parents can promote PA among their preschoolers, not only by limiting TV time but also by being highly supportive of their children's active pursuits.

## 1. Introduction

It is widely accepted that physical activity has numerous positive health outcomes including its influence on meeting healthy weight goals, when associated with low-energy intake through healthy eating habits [1–4]. In children, physical activity is particularly important as it improves gross and fine motor skill development necessary for academic performance (e.g., writing), self-perceived competence (academic as well as athletic) as well as increasing socioemotional adjustment and self-esteem [5]. Physical activity in groups and games also have social benefits in that they offer children opportunities to learn new skills [6] while developing friendships [7].

Physical activity is defined as any physical movement resulting from skeletal muscle contraction [8]. In contrast, sedentary activities can include watching TV/videos, playing video games, computer time, and reading. According to Social Learning Theory [9], individuals learn their habits and attitudes toward PA very early in their development, by observing and imitating their parents. According to Welk, Wood, and Morss [10], there are two aspects of parental behaviours that promote PA in children: (1) role modeling, which includes a parent's interest in PA as well as their efforts to be active, and (2) parental support, which refers to parental encouragement, involvement (i.e., participating in PA with the child), and facilitation such as providing access and opportunities for the child to be active (e.g., transportation to arenas and parks).

Supporting the importance of parental support and role modeling in their children's PA habits, research suggests that there is a link between parental PA [11], encouragement, involvement/interaction, support [12–14], and their children's PA. Moore et al. [11] found that children between 4 and 7 years of age were 3.5 to almost 6 times more likely to be active when one or both parents were active than when both parents were inactive. Among the various components of parental influence, it appears that parental facilitation exerts the greatest independent influence on young children's PA [10]. In addition, there is evidence that parental support of child PA contributes to the maintenance of PA habits later in adolescence, at least in girls [15].

Researchers have suggested that the benefits of PA on health are moderated by sedentary behaviors; however, findings are inconsistent. For example, Proctor at al. [16] and Janz et al. [17] found that children who watched the most TV and who were the least physically active had the greatest increases in body fat from preschool to early adolescence whereas children who were active and watched little TV gained the least. On the other hand, Hinkley et al.'s [12] literature review found that about half of the studies examined reported no association between PA and sedentary behaviours. As such, there is an ongoing need for more research in this area, if only to better understand the relationship between PA and TV viewing in young children. 

As with any other type of learning, parental role modeling does not entirely explain children's development of PA habits. Research has also demonstrated the salience of personal, familial, and environmental factors that influence both physical activity and sedentary behaviors in children. Among personal factors, a number of authors have found that boys are more active [12, 18], and that they engage in more vigorous activity than girls [19, 20]. There is also an age-effect among preschool children, with some studies indicating reduced physical activity levels between the ages of 4 to 5 years compared to during the third year of life, for both boys and girls [20, 21]. Weight status does not seem related to the activity levels of preschoolers according to a review by Hinkley et al. [12]. 

Among familial characteristics that influence children's physical activity are parents' education level and income [12, 18, 22]. Little is known about the influence of parents' marital status on activity and the sedentary habits of their young children. According to Hinkley et al. [12], single-parent status has not been examined as a potential correlate of PA in preschoolers. Time spent outdoors as well as access to play areas and facilities are two of the most commonly investigated physical environmental factors associated with PA and both have been positively associated with increased levels of PA among preschool-aged children [12, 18, 23, 24]. 

Despite the aforementioned predictors of early development of PA habits, there are very few studies conducted with very young children. Timmons et al. [23] suggested that the underrepresentation of preschoolers in study sampling may be based on the erroneous assumption that young children are sufficiently physically active. These misperceptions might be explained by the lack of specific physical activity recommendations for children under 6 years of age, as is the case in Canada [25]. In general, it is recommended that preschoolers accumulate at least 60 minutes of structured (organized) PA and 60 minutes of unstructured (informal) PA per day [26] and that TV viewing be limited to one hour per day for preschoolers [27]. Research shows that Canadian children between the ages of 2 and 11 years watched an average of 2 hours of TV per day [28]. It has been shown that preschoolers do not typically engage in PA for consecutive minutes; instead, they tend to engage in brief bouts of movement and spend little time at vigorous intensity levels [23]. Other researchers have demonstrated that preschoolers spend most of their free-play or unstructured time engaged in sedentary activities [29, 30]. When they are not idle, researchers have found that their activity levels do not meet recommended thresholds. Rather, they are most likely to engage in only 32 minutes of activity during 2-hour stints of outdoor playtime [30]. In short, findings have not supported the commonly held belief that preschoolers are highly active.

It would appear that no research has examined the combined effect of parental influence as well as child and family characteristics on the PA and sedentary behaviors of preschool children. Moreover, few efforts have targeted parental attitudes toward PA, particularly in samples of very young children. This is unfortunate since parental enjoyment of PA is likely a strong determinant of such in their children. Also, no studies have considered parental perceptions of their preschoolers PA. Consistent with findings that a large proportion of parents fail to recognize overweight or obesity in their children [31–33], parents who perceive that their child's level of PA is sufficient are less likely to apply what they have learned in prevention programs regarding eating behaviours and physical education [32]. Therefore, measuring parents' perceptions of their child's PA levels is relevant. 

With this in mind, the main objective of this study was to examine the influence of parents on their children's PA. The links between their enjoyment of PA, the degree of importance they assign to their child's PA abilities and their support of PA habits on their preschool-aged children are also explored. A secondary objective was to determine which child characteristics (e.g., age, gender, TV/Video viewing, weight status and linguistic group) and parent characteristics (e.g., income, education, age, marital status and weight status) best predict PA.

## 2. Method

### 2.1. Procedure

This study is a part of a larger project conducted between January and September 2008, which examined individual and family factors associated with preschool children's overweight and obesity levels using parent, child, and teacher interviews and questionnaires. The BMI was calculated for participants and served as an indicator of subjects' levels of overweight and obesity. Consent was sought for all aspects of the study.

Twenty-six licensed, centre-based, child care facilities in the City of Greater Sudbury (Ontario, Canada) were contacted. The centres were selected from different neighborhoods within the city and surrounding areas to provide a sample that represented the broad range of socioeconomic, ethnic and linguistic (primarily French and English) backgrounds found in the region. When the authorization of the day cares were obtained, the research assistants distributed consent forms along with a letter explaining the study to parents of all children between the ages of 3 and 5 years at each of the participating child care centres. One hundred and two parent-child dyads gave their consent for themselves and their child to be interviewed. They were also asked to give their consent for the daycare worker to answer a questionnaire about their child's physical activity. Parents received $10 for their participation. The study was approved by the university's Research Ethics Board where the authors are employed.

### 2.2. Participants

Fifty-four boys (52.9%) and 48 girls (47.1%) between 3 and 5 years of age (M = 3.75 years; SD = .80) and their parents (mean age = 34.0 years; SD = 7.0) participated in this study. Forty-nine children were three years of age (46.7%), 33 were four years of age (31.4%), and 23 were five years of age (21.9%). An equal number of child-parent dyads were recruited from French and English-speaking child care centres (*n* = 53). Almost all of the parents responding to the questionnaires were women (*n* = 98). Only 4 parent respondents were men. The majority of parents were married (*n* = 74, 72.5%), while approximately one-quarter were single, separated, or divorced (*n* = 28, 27.5%). Family income level was categorized as low ($49 999 or less), middle ($50 000 to $74 999), and high ($75 000 and above). Nearly half of the families had an annual income of $75 000 or more (*n* = 46, 47.9%) while 21 (21.9%) and 29 (30.2%) were classified in the middle and low-income groups, respectively. Thirty-nine parents (38.6%) reported that they had completed postsecondary education.

### 2.3. Instrument

The questionnaires were designed specifically for the purpose of this study. Some questions and variables were adapted or taken from pre-existing questionnaires while others were created by the research team. A bidirectional translation method was used to translate the questionnaires from English to French (i.e., the questionnaires were translated then verified by members of the research team). The questionnaires were pretested by adults in both languages. The questionnaires administered to the parents sought child characteristics (e.g., age and gender), the average amount their child watches TV or videos each day, the child's daily physical activity (PA), their perception of the intensity of their child's PA, parent characteristics (e.g., gender, marital status, household income, and level of education), and parental PA behaviours and attitudes. The items selected for the study are described in the following paragraphs.

### 2.4. Dependent Variables

#### 2.4.1. Child Daily PA

Parents were asked to indicate the total amount of time, on average, their child participated in daily physical activity (e.g., sports) and/or physically active play (e.g., playing tag or climbing on a gym set). The items were rated on a four-point Likert scale as follows: (1) “less than 30 minutes per day”, (2) “30 to 60 minutes per day”, (3) “60 to 120 minutes per day”, and (4) “120 minutes or more per day”. As per PA recommendations [26], child daily PA was then dichotomized into either one hour or more of PA per day or less than one hour of PA per day. Three quarters of the children (75.5%) participated in at least one hour of daily PA; however, considerably more boys (83.3%) than girls (66.7%) achieved the recommended duration of PA, *χ* ^2^ (1, *N* = 102) = 3.82, *P* < .05.

#### 2.4.2. Parent's Perception of Intensity of Child Activity

In addition to the amount of time children spent engaged in daily physical activity, parents were asked to rate their perception of the intensity of their child's activity at different times during the day: before breakfast, morning, early afternoon, late afternoon, and after dinner. A five-point Likert scale was used: (1) “not at all active”, (2) “slightly active”, (3) “moderately active”, (4) “very active”, and (5) “highly active”. An overall average activity level was computed for each child from these five items then categorized into three groups as follows: inactive (an average score < 3.0), moderately active (an average score ≥ 3.0 and < 4.0), and highly active (an average score ≥ 4.0). The majority of children were considered moderately active (58.8%). Another 14.7% were considered highly active and about one quarter of the sample (26.5%) was deemed inactive. No significant gender differences were found among the three groups *χ* ^2^ (2, *N* = 102) = .55; *P* = .76.

To assess the criterion concurrent validity of this measure [34], daycare workers were asked to report on their perceptions of the activity level of each child. The questions for daycare workers were similar to those used with parents but included only three time periods (morning, early afternoon, and late afternoon) to reflect the timeframe when children were typically at the centres. The scale was identical to that used for the parent questionnaire. The mean for the 3-item scale was computed and compared to the mean rating reported by parents using a paired samples *t*-test. The results revealed that perceived child activity level ratings did not significantly differ between parents and daycare workers, *t*(98) =.28, *P* = .78, demonstrating good concurrent validity in this sample. For both measures (parents and teachers) the Cronbach alpha reliability coefficients were moderate, that is  .64 and  .67, respectively. Higher scores were not expected as physical activity naturally varies during the day according to the schedule (period of structured inactivity at school, mealtime, or other activities at home).

### 2.5. Measures of Predictor Variables

#### 2.5.1. Child Characteristics

Parents completed a questionnaire that sought their child's date of birth, gender, and the time spent by their child watching TV or videos each day (“TV time”). The Canadian Paediatric Society [27] recommends that TV viewing for preschoolers be limited to one hour per day. Based on this guideline, TV time was simply dichotomized as one hour of TV time or less per day or more than one hour of TV time per day. Only half the children (45.4%) in the study engaged in more than one hour of daily TV time. No significant gender differences in the amount of TV time were observed, *χ* ^2^ (1, *N* = 99) = .14, *P* = .43.

#### 2.5.2. Child Weight Status

Weight status for each child was determined using the body mass index (BMI). Each child was classified in one of the two following categories: “underweight/normal weight” (< 85th percentile), or “at-risk of overweight/overweight” (≥ 85th percentile) [35]. Overall, twenty-eight children (28.0%) were either overweight or at-risk of overweight and 72 children (72.0%) were considered normal weight. There were no significant gender differences in the proportion of children who were overweight/at-risk of overweight and those of normal weight, *χ* ^2^ (1, *N* = 100) = .16; *P* = .43.

#### 2.5.3. Parent Characteristics

Parents reported their age, gender, marital status, household income and level of education. Similar to the protocol for children, parents' height, and weight were measured by the researchers and used to calculate their BMI. The mean BMI for the parents was 27.0 (SD = 6.01). According to the Center for Disease Control and Prevention [35], about half of the parents in the study were either overweight (23.3%) or obese (30.2%). None of the parents were underweight and 46.5% were classified as normal weight.

#### 2.5.4. Parental Physical Activity Behaviours and Attitudes

Parents reported on their physical activity behaviours and attitudes. The items were drawn from scales used in the Amherst Health and Activity Study [14]. The variables included parents' support for children's PA, parents' PA (i.e., role modeling), parents' enjoyment of PA, and the importance parent's placed on the child's PA abilities.

#### 2.5.5. Parental Support for PA

Five questions assessed how often during a typical week adults in the household provided the child with support for physical activity (i.e., encouraged the child to be active, participated in PA with the child, provided transportation for the child to go somewhere to be active, watched the child engage in PA, or told the child that PA was good for his/her health). A five-point Likert scale consisted of the following: (0) “none”, (1) “once”, (2) “sometimes”, (3) “almost daily”, and (4) “daily”. The responses from each adult for the five items were averaged and a combined parent/adult mean score was then computed (M = 2.81, SD = .69). The internal consistency of “parental support for PA”, measured by Cronbach's alpha, was 0.75. This finding was similar to that obtained by Trost and colleagues [14], 0.78, in a study that used the same scale with parents of older children (grades 7 through 12).

#### 2.5.6. Parental PA

A three-item measure assessed the frequency of the parents' physical activity habits. Parents were asked how often in a typical week they “walked for exercise”, “participated in sporting activities for at least 20 minutes that made them sweat and breathe hard”, and “did heavy house cleaning, gardening or yard work for at least 20 minutes at a time”. Similar to the “parental support” measure, an average combined score from the parents was computed for the “parental PA” variable (M = 2.80, SD = 1.26).

#### 2.5.7. Parental Enjoyment of PA

With a single item on a five-point Likert scale ranging from (1) “not enjoyable” to (5) “very enjoyable”, parents were asked to indicate how much the adults in the family “enjoy physical activity or exercise”. A combined average score for the parents was calculated (M = 4.19, SD = .84).

#### 2.5.8. Importance of Child's PA Ability

Parents also reported how important it was to each adult in the household that the child was “good at sports and physical activities” on a five-point Likert scale ranging from (1) “very unimportant” to (5) “very important”. Again, the scores from the adults were averaged to create a composite score (M = 3.54, SD = 1.20).

## 3. Results

### 3.1. Statistical Analyses

All statistical analyses were performed using the Statistical Package for the Social Sciences (SPSS Version 15.0). Logistic and multiple regression analyses using child characteristics, parent characteristics, and parents' physical activity behaviours to predict the reported child daily activity and the parent's perceived intensity of a child's physical activity were performed.

### 3.2. Predicting Children's Daily PA

Logistic regressions were performed to predict child daily PA from child characteristics (first model), parent characteristics (second model), and parental PA behaviours (third model). The results are shown in Table 1.

The test of the full model predicting child daily PA with all child characteristic predictor variables was significant, *χ* ^2^ (5, *N* = 97) = 18.4; *P* < .01, with a goodness of fit of *χ* ^2^ (8, *N* = 97) = 8.04, *P* = .43. Overall, 83.5% of the cases were accurately predicted. Of the child characteristics, only watching less than an hour of TV/videos per day significantly predicted whether children participated in the recommended amount of daily physical activity (one hour). In fact, children who watched less TV were 4.7 times more likely (*B* = 1.47, *P* < .01) to participate in sufficient PA. However, it should be noted that children's age and gender approached significance levels. Specifically, male children were almost 3 times more likely to meet recommended levels than girls (*B* = 1.08, *P* < .10) and children were slightly less likely to be active for at least an hour a day with each month increase in age (*B* = −.04, *P* < .10). 

In the second model, the test of the full model predicting child daily PA from parent characteristic predictor variables (e.g., age, marital status, income, and education) was not found to be significant *χ* ^2^ (5, *N* = 96) = 4.82, *P* = .44. The goodness of fit obtained was *χ* ^2^ (7, *N* = 96) = 13.1, *P* = .07.

For the third model predicting child daily PA from parental PA predictors, the test of the full model was significant, *χ* ^2^ (4, *N* = 99) = 23.1; *P* < .01, with a goodness of fit of *χ* ^2^ (8, *N* = 99) = 7.28; *P* = .51. Overall, 77.8% of the cases were accurately predicted. Among the four parental PA variables, only parental enjoyment of PA significantly predicted child daily PA, whereas parent's support and PA habits approached statistical significance. The more parents supported their child's activity (*B* = .780, *P* < .10) or the more active the parents (*B* = .482; *P* < .10) or the greater their enjoyment of PA (*B* = .697; *P* < .05), the more likely the children were to engage in the recommended amount of daily activity. The importance parents placed on their child's PA abilities did not predict the outcome variable of interest.

### 3.3. Predicting the Parents' Perceived Intensity of a Child's PA

A multiple logistic regression analysis was performed to predict child PA intensity level from child and parent characteristics and parental PA behaviours. Child PA intensity levels were categorized into three groups: inactive, moderately active, and highly active. The results are presented in Table 2 with the highly active and moderately active groups displayed and the inactive group shown as the reference category. Similar to prior logistic regression analyses conducted in this study, child characteristics were used in the first model, parent characteristics in the second model, and parental PA behaviours/attitudes in the third model. 

The test of the full model predicting child PA intensity levels using child characteristic predictor variables was significant, *χ* ^2^ (10, *N* = 97) = 20.3; *P* < .05, with a goodness of fit of *χ* ^2^ (166, *N* = 97) = 142.2; *P* = .91. Overall, 66.0% of the cases were accurately predicted. Only child age significantly predicted PA intensity level with older children being slightly less likely to be highly active (*B* = −.11; *P* < .01) or moderately active (*B* = .08; *P* < .01) than inactive for each month increase in age. As shown in Table 2,none of the other child variables such as gender, linguistic group, TV time, or weight status significantly predicted PA intensity levels. 

In the second model, the test of the full model predicting child activity level from the parent characteristics versus the constant-only model was also found to be significant, *χ* ^2^ (10, *N* = 96) = 23.9, *P* < .01, with a goodness of fit of *χ* ^2^ (112, *N* = 96) = 102.4, *P* = .73. Overall, 61.5% of the cases were accurately predicted. Parent age was found to be the only significant predictor. Children of older parents were less likely to be highly active (*B* = −.27; *P* < .01). Marital status, parent education, and household income did not significantly predict child PA intensity level in this sample.

For the third model, the test of the full model predicting child activity level from parental PA behaviours versus the constant-only model was significant, *χ* ^2^ (8, *N* = 99) = 19.6; *P* < .05, with a goodness of fit of *χ* ^2^ (186, *N* = 99) = 164.6; *P* = .87. In this model, 60.6% of cases were accurately predicted. Among the parental PA behaviour variables, parental support for PA and parental PA significantly predicted child PA intensity level. Children of parents who provided greater support for PA were over 4 times more likely to be highly active (*B* = 1.44; *P* < .05). Greater parental PA also increased the likelihood that children would be highly active (*B* = .68, *P* < .05) almost two-fold.

## 4. Discussion

Using a representative sample of Canadian parents, the current study examined the variables that best predicted the PA behaviours of their children. While social learning theory predicts a strong link between parental role modeling of PA and the performance of this behaviour by their offspring, the extant literature has insufficiently considered the relative influence of other predictors of PA within the same study including parental support for PA, parental enjoyment of PA, and the importance parents assign to their children's PA abilities. Moreover, research of this nature has largely overlooked younger age groups of children. With this in mind, parental influence on the PA habits of 102 male and female preschool-aged children was examined. In addition to direct parental influence, this study also considered the effect of child characteristics (e.g., weight status, the amount of television watched) and adult characteristics (e.g., age, marital status, income, education, attitudes) on the intensity and frequency of PA. The discussion that follows below presents the reader with an overview of the study's main findings, while highlighting the particular relevance of parental habits, parental support of PA, and parental enjoyment of PA on their children's PA.

By way of summary, approximately three-quarters of the children in this study were reported to participate in at least 1 to 2 hours of PA per day. This seemingly high-participation rate can be explained in part by policies that oblige Canadian child care centres to provide children at least two hours each day (weather permitting) of outdoor play to children over the age of thirty months, [36]. Parents may be aware of these standards and assume that their child is participating sufficiently in PA. However, while it may seem that preschoolers engage in sufficient activity, previous researchers have pointed out that the majority of time spent in these play periods are spent in sedentary or light activity [29, 30]. Thus, children may actually be getting much less exercise than their parents believe if they rely solely on day cares to provide such. Of concern is that approximately 27% of children in the current study were considered by parents as generally inactive. 

While the majority of children in the current study met the one-hour requirement of daily PA (75%), almost half of the sample (45%) watched more than the one-hour maximum of TV viewing per day recommended by the Canadian Pediatric Society [27]. Moreover, TV watching emerged as one of the strongest predictors of child daily PA. On the other hand, and contrary to previous research showing that TV watching is associated with higher BMI in preschool children [16, 37], we did not find that a child's weight associated itself strongly with their daily PA. This appears to be in line with research by Jago and colleagues [37], who noted that the association between TV watching and BMI emerges only at around 6 years of age. All of the participants in the current study were below 6 years of age and therefore it may be that children in this study were still too young for this association to have emerged. 

It was found that different sets of factors predicted child daily PA (binary logistic regression) and child activity levels (multinomial regressions). TV time and parents' enjoyment of PA were the only significant predictors of children's daily PA whereas age (child and parent), parental support of PA, and PA habits predicted children's membership in two of three categories of perceived intensity of PA; that is, highly active or moderately active. Because child daily PA is a measure of the amount of time a child spends engaged in physical activities and the child's PA levels is a qualitative measure of PA, it is possible that parents used different criteria to assess these two components. In other words, it may be the case that parents estimated their child's daily activity on the basis of their knowledge of the child's routine. It follows that related measures such as TV time and enjoyment of PA (which is likely to be associated with families' PA and leisure time) predicted the child's PA. On the other hand, parents' assessment of the child's level of PA may depend on their perception of the child's level of development (younger children requiring more supervision and care might be perceived as more active), and perception of their supportive behavior of PA including their own level of PA.

Results showing that less time watching TV predicted the recommended amount of daily PA might simply illustrate the point that time spent watching TV leaves less time for children to be physically active. On the one hand, it is unlikely that parents would intentionally want their child to be sedentary instead of active. Rather, parents might encourage more TV time in their young children because, as reported by He and colleagues [38], they use TV as a coping tool and babysitting technique so that they can tend to household chores such as cooking and cleaning. On the other hand, studies have also pointed out that the same child can be both highly sedentary and highly active [39]. The results from multiple regression analyses for children's PA intensity levels support this possibility. In short, TV time did not significantly predict whether children were highly active or moderately active versus inactive, despite the fact that it influenced the amount of children's daily PA. Thus, it seems plausible that some of the children in this study watched a lot of TV but were still considered highly active by their parents. Conversely, children who watched very little TV may still have been considered inactive if they spent a lot of their time in other sedentary pursuits such as playing with toys, puzzles, or doing arts and crafts.

Among measures of parental attitudes, only the importance they confer to their child's PA abilities failed to predict child daily PA or PA intensity levels. It is expected that a parent will naturally wish a child to gain new skills and abilities and to be successful in any activity he or she may engage in. However, parents might not see the importance of ability for children who are that young or they may simply accept that such abilities develop later. Parental enjoyment of PA, their PA habits as well as the support they offer their children in their pursuit of PA were cogent predictors and certainly underline the importance of social learning.

Child age and gender were marginally significant predictors (*P* < .10) of daily PA. Older children were slightly less likely to get sufficient PA and boys were almost 3 times more likely to engage in at least an hour or more of PA per day. The results parallel existing research showing that boys are consistently more active [12, 18], and engage in more vigorous activity than girls [19, 20]. Younger children are also more active and less sedentary [20, 21]. Using multiple regression analyses to predict PA intensity levels, older children were significantly less likely to be identified as highly active compared to inactive. Together, the findings of a decrease in activity with an increase in age may be largely explained by the fact that older preschool children are more likely to spend more time at the child care centre preparing for the transition toward more academics in the elementary school setting. Pate et al. [40] suggested that as children get closer to full-time school attendance, the structured, preacademic activities of the older preschool child's classroom outweigh the free play typically seen in classrooms of younger children. 

The current study is one of the few in the extant literature that has scrutinized parental influence of PA behaviours (both mother and father) on children's PA habits. The majority of studies on childhood PA have focused on school-aged children while few have focused on the PA patterns of preschoolers. This developmental period is key in the foundation of healthy habits such as being active on a regular basis. Thus, investigating factors that can encourage children to be active from a young age is an important component in combating childhood obesity. Finally, very few studies have looked at the importance parents place on their child's PA ability, particularly in this young age group. This particular variable was not found to be an important predictor of children's PA and an unlikely candidate for further inquiry.

Some results should be interpreted with caution. First, the cross-sectional design does not permit causal inferences between PA outcome variables and predictor variables. It remains unclear if parents who provide a highly supportive environment for their child to be active cause the child to become more active or if it is an active child that influences the degree to which parents provide support for his or her active pursuits. Another limitation of the study was the use of self-report methods to assess child PA rather than using objective measures such as direct observation or accelerometry. The costs of such methods were prohibitive for the current study; however, a number of steps were taken to strengthen the validity of the findings. For instance, we asked both parents and daycare workers to report on two aspects of child PA—their daily amount of PA and each child's overall activity level for different timeframes throughout the day. This provided a more comprehensive understanding of preschool children's overall PA habits. Chen and colleagues [41] found that nursery school teacher's ratings of child activity levels, a measure that was similar to that used in the current study, was a valid method for assessing child PA when compared with two objective monitoring devices. Further, Noland and others [42] examined the validity of the same measure of child activity level used in this study and found a moderate correlation between parent- and teacher-reported child activity levels. Since comparisons between the parent and daycare worker activity level scores in this study did not significantly differ, we can conclude that the PA assessment measures were reasonably accurate reflections of children's PA habits.

## 5. Conclusion

In summary, parents occupy a privileged position in terms of influencing their children's physical activity. First, they are the custodians of daily schedules and can therefore guide issues such as the amount of television viewed. They also have a voice in their children's education and can help ensure day cares follow legislated requirements for quality, daily PA. Second, parents have a direct influence on their children's PA. Their support of PA, their own level of PA, and their enjoyment of PA predict the extent to which their young will engage in PA with sufficient intensity and duration. Far less important, based on this study, is the degree to which parents feel that their children have well-developed abilities when they perform PA.

Results of this study suggest that additional attention should be paid to girls who were found to be less active than their male counterparts. This is certainly consistent with previous research. Similarly, parents may wish to accentuate their support of PA as their children age since it appears that they are less active with each month of development. Whereas being an older parent is a negative correlate of children's PA, marital status, language, educational levels, and household income were not. 

As there is evidence that excess weight can track throughout childhood into adulthood [43, 44], public health officials should consider these findings and incorporate appropriate strategies for the prevention of obesity and promotion of PA among young children. Key is the involvement of parents in their children's well-being. As has been made clear in previous research, intervention and prevention efforts beginning early in childhood may also benefit the children's cognitive and socioemotional development in enhancing self-perceived competence and learning through fine and gross motor skills development as well as increasing their social skills through organized sports and physical activities.

## Figures and Tables

**Table 1:** Logistic regression predicting child daily PA.

Predictors	Beta (SE)	Wald	Odds ratio	Chi-Square
				*χ* ^2^
Child Age (months)	−.044 (.024)	3.58	.956*	
Child Gender (male)	1.075 (.552)	3.78	2.93*	
Linguistic Group (French)	.382 (.567)	.454	1.465	
TV Time (1 hr or less/day)	1.555 (.562)	7.65	4.74***	
Child At-risk of Overweight/Overweight	.171 (.620)	.076	1.187	18.37***
Parent Age (years)	−.034 (.034)	1.010	.966	
Married	−.869 (.695)	1.567	.419	
Income 0–45 K	−.560 (.745)	.565	.571	
Income 45–75 K	−.029 (.746)	.001	.972	
Postsecondary Education	.305 (.723)	.178	1.356	4.82
Parental Support for PA	.780 (.432)	3.25	2.18*	
Parental Enjoyment of PA	.697 (.341)	4.19	2.01**	
Parental PA Habits	.482 (.251)	3.69	1.620*	
Importance of Child's PA Ability	−.034 (.260)	.017	.966	23.1***

*P < .10; **P < .05; ***P < .01.

**Table 2:** Multinomial logistic regression predicting parents' perception of their child PA intensity level.

	Highly Active			Moderately Active			

Predictors							Chi-Square
	Beta (SE)	Wald	Odds Ratio	Beta (SE)	Wald	Odds Ratio	*χ* ^2^
Child Age (months)	−.113 (.038)	8.73	.893***	.079 (.023)	11.39	.924***	
Child Gender (male)	.676 (.772)	.767	1.967	.116 (.516)	.050	1.123	
Linguistic Group (French)	.325 (.747)	.190	1.384	.204 (.532)	.147	1.226	
TV Time (1 hr or less/day)	.303 (.749)	.163	1.354	−.283 (.523)	.420	1.404	
Child At-risk of Overweight/Overweight	−.064 (.798)	.006	.938	.339 (.565)	.251	.754	20.3**
Parent Age (years)	−.266 (.095)	7.81	.766***	−0.19 (.038)	.252	.981	
Married	.808 (.985)	.672	2.243	.193 (.670)	.083	1.213	
Income 0–45K	.611 (1.205)	.257	1.842	.134 (.764)	.031	1.144	
Income 45–75K	−.259 (1.052)	.061	.772	−.756 (.618)	1.497	.469	
Postsecondary Education	−1.486 (1.378)	1.164	.226	−.165 (1.132)	2.125	.192	23.9***
Parental Support for PA	1.441 (.733)	3.86	4.22**	.478 (.376)	1.614	1.613	
Parental Enjoyment of PA	.458 (.548)	.699	1.58	.080 (.300)	.071	1.083	
Parental PA Habits	.680 (.315)	4.67	1.974**	.227 (.217)	1.09	1.255	
Importance of Child's PA Ability	−.145 (.306)	.225	.865	.066 (.228)	.085	1.069	19.56**

*P < .10; **P < .05; ***P < .01.
